# Supplementary material for: An ancestral SARS-CoV-2 vaccine induces anti-Omicron variants antibodies by hypermutation
Source: Nat Commun. 2024 Apr 20;15:3368. doi: 10.1038/s41467-024-47743-1 (PMC11032360; doi:10.1038/s41467-024-47743-1)
Supplement: Supplementary file 1 — Supplementary Information [file 41467_2024_47743_MOESM1_ESM.pdf]

Supplementary information

An ancestral SARS-CoV-2 vaccine induces anti-Omicron variants antibodies by hypermutation

Seoryeong Park[1,2*], Jaewon Choi[3,4*], Yonghee Lee[5], Jinsung Noh[5,6], Namphil Kim[5], JinAh Lee[7], Geummi Cho[1,8], Sujeong Kim[1,8], Duck Kyun Yoo[1,8], Chang Kyung Kang[9], Pyoeng Gyun Choe[9], Nam Joong Kim[9], Wan Beom Park[9], Seungtaek Kim[7], Myoung-don Oh[9], Sunghoon Kwon[3,5,6], Junho Chung[1,2,8]

Supplementary Figures 1-11

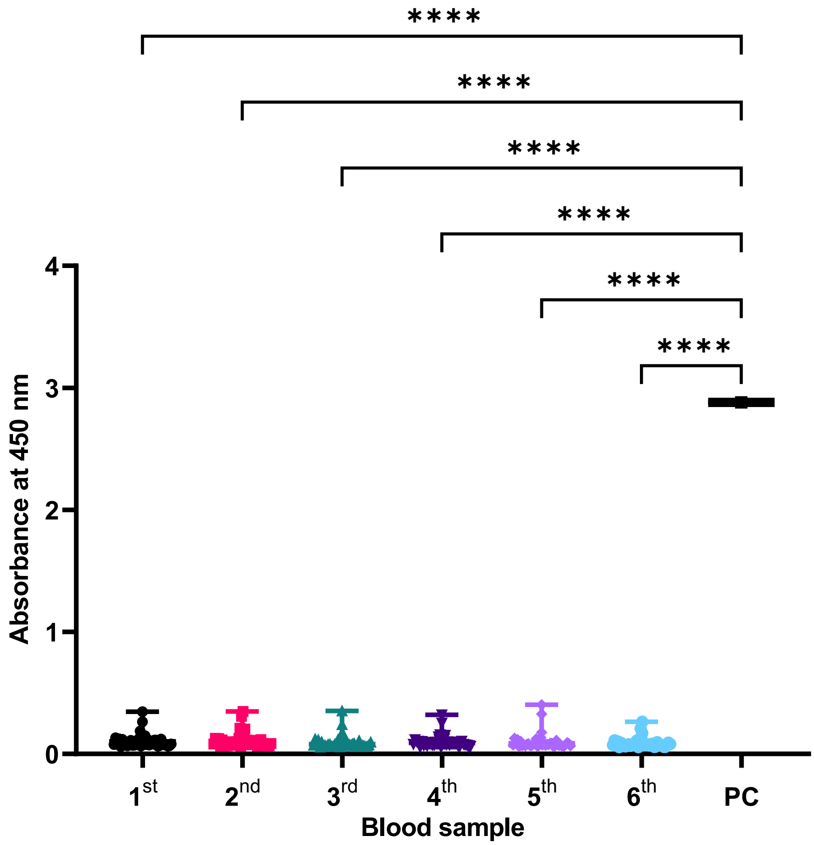

Supplementary Fig. 1 Plasma levels of antibodies against the SARS-CoV-2 N protein. Reactivity of plasma from 41 vaccinees and a COVID-19 patient (Positive control; PC) to the SARS-CoV-2 N protein (Sino Biological) was tested by ELISA. The P value was calculated using one-way ANOVA. ****, $p < 0.0001$. All box plots include the median line, the box denotes the interquartile range (IQR), whiskers denote the rest of the data distribution.

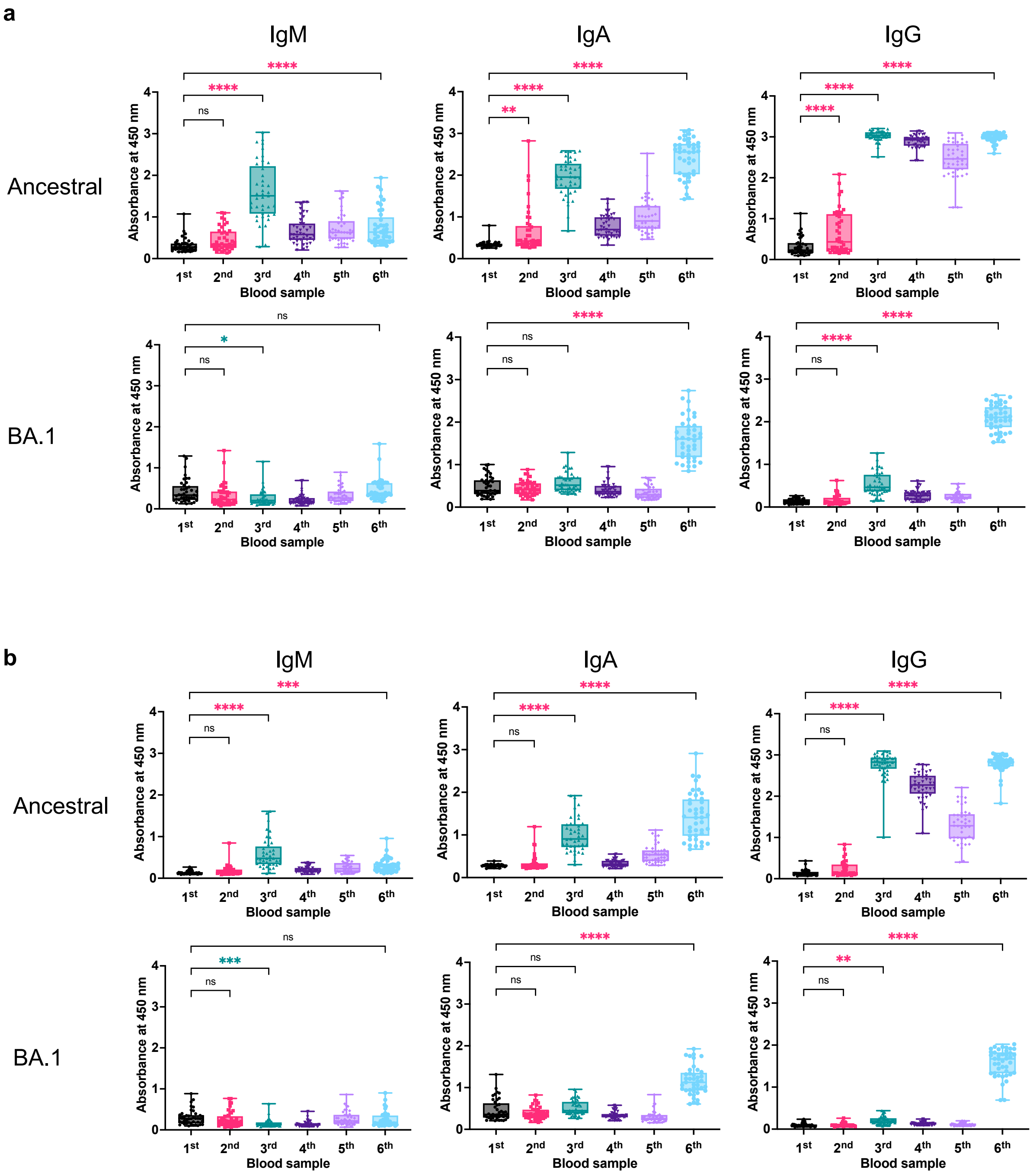
a
IgM
IgA
IgG
Ancestral
BA.1
Absorbance at 450 nm
Blood sample
1st
2nd
3rd
4th
5th
6th
ns
*
**
***
****
b
IgM
IgA
IgG
Ancestral
BA.1
Absorbance at 450 nm
Blood sample

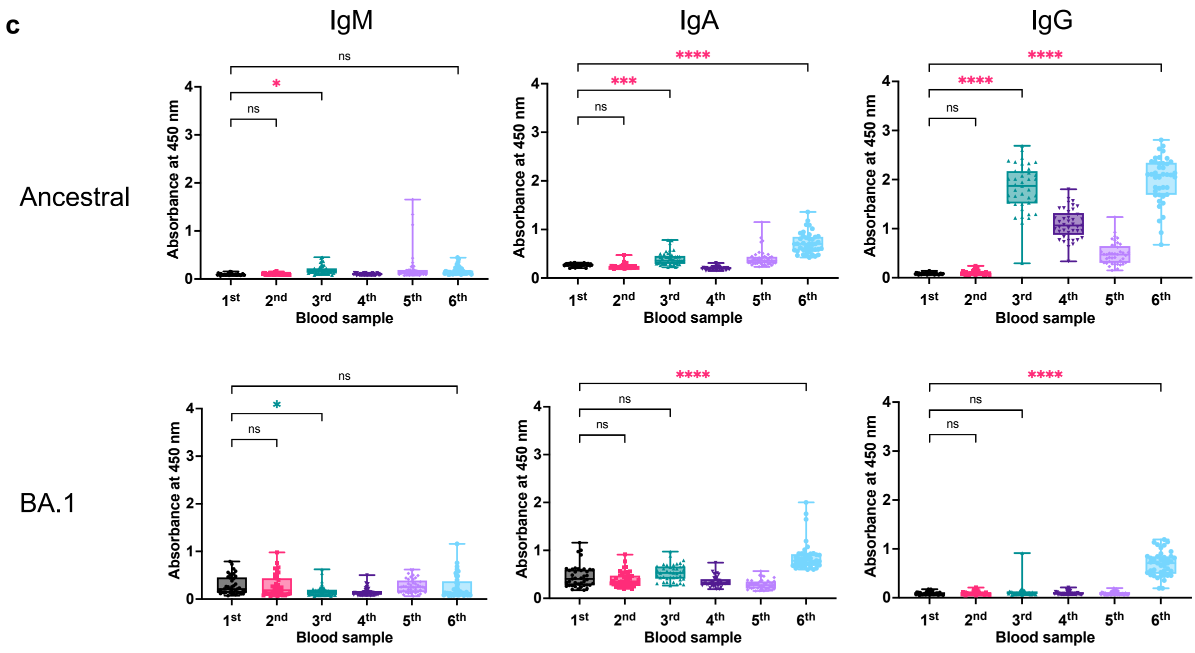

Supplementary Fig. 2 Plasma levels of antibodies against ancestral and BA.1 RBD. Plasma levels of antibodies to SARS-CoV-2 ancestral and BA.1 RBDs were measured in 41 vaccinees after (**a**) 100-, (**b**) 500-, or (**c**) 2,500-fold dilution. All experiments were performed in duplicate, and the average value for each vaccinee was plotted. The P value was calculated using one-way ANOVA. ns, not significant, $p > 0.05$; **, $p < 0.01$; ***, $p < 0.001$; ****, $p < 0.0001$. The increases and decreases in antibody levels are marked with red and green asterisks, respectively. All box plots include the median line, the box denotes the interquartile range (IQR), whiskers denote the rest of the data distribution. Source data are provided as a Source Data file.

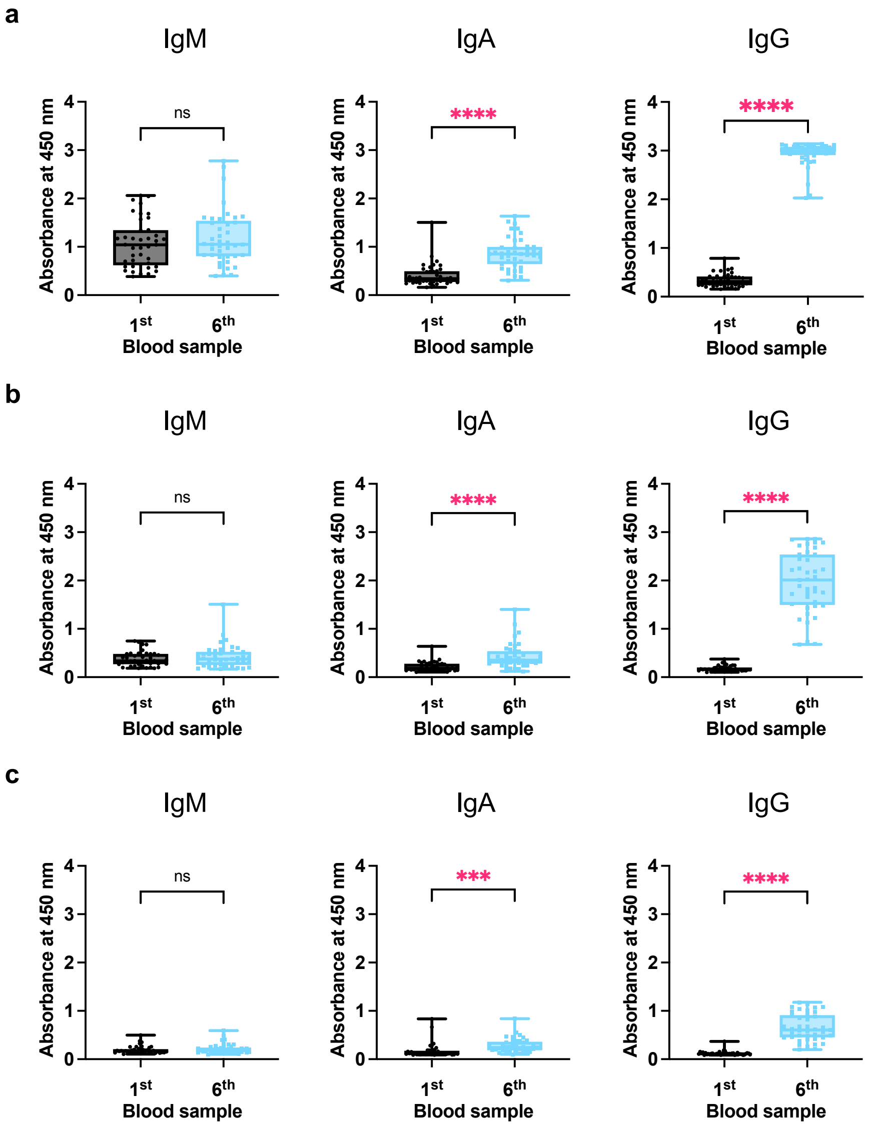

Supplementary Fig. 3 Plasma levels of antibodies against the RBD of Omicron subvariant BQ.1.1. Plasma levels of antibodies to the Omicron subvariant BQ.1.1. RBD (Sino Biological) were determined after (**a**) 100-, (**b**) 500-, or (**c**) 2,500-fold dilution. All experiments were performed in duplicate, and the average value for each vaccinee was plotted. The P value was calculated using one-way ANOVA. ns, not significant, $p > 0.05$; ***, $p < 0.001$; ****, $p < 0.0001$. All box plots include the median line, the box denotes the interquartile range (IQR), whiskers denote the rest of the data distribution. Source data are provided as a Source Data file.

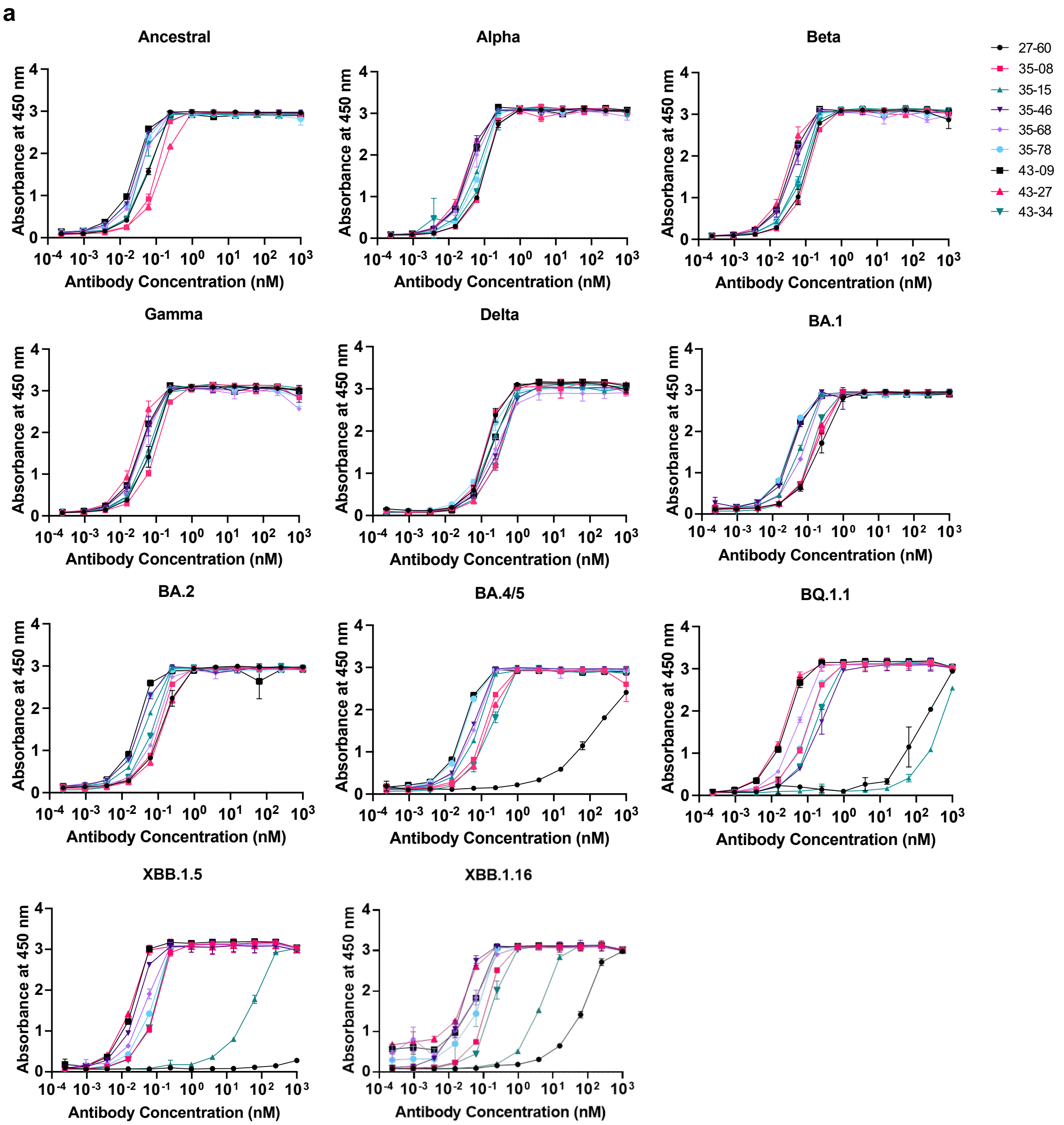
a
Ancestral
Alpha
Beta
Gamma
Delta
BA.1
BA.2
BA.4/5
BQ.1.1
XBB.1.5
XBB.1.16
Absorbance at 450 nm
Antibody Concentration (nM)
27-60
35-08
35-15
35-46
35-68
35-78
43-09
43-27
43-34

b

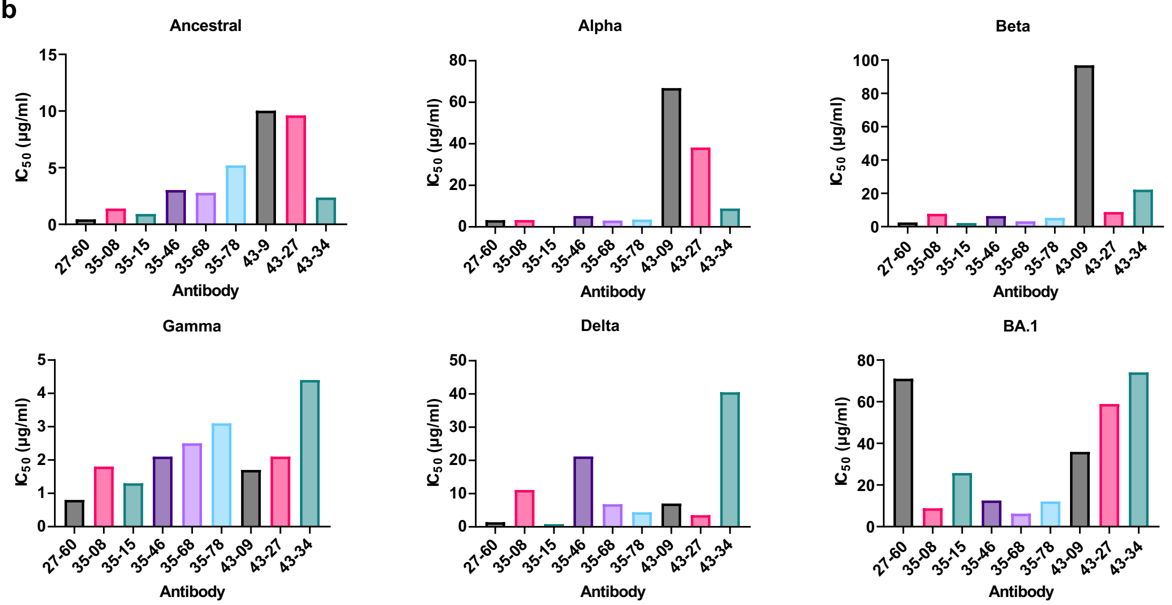

Supplementary Fig. 4 Characteristics of BA.1 RBD-reactive clones. **a,** Reactivity of BA.1 RBD-reactive clones from the phage display library was tested against SARS-CoV-2 ancestral, Alpha, Beta, Gamma, Delta, and Omicron-sublineage (BA.1, BA.2, BA.4/5, BQ.1.1, XBB.1.5, and XBB.1.16) RBD proteins in the form of a recombinant scFv-hFc-HA protein. All experiments were performed in duplicate, and the data are presented as the means ± SD. **b,** The neutralization efficacy of BA.1 RBD-reactive clones was tested in a microneutralization assay using ancestral SARS-CoV-2 (βCoV/Korea/KCDC03/2020 NCCP43326), Alpha B.1.1.7 (hCoV-19/Korea/KDCA51463/2021 (NCCP 43381), Beta B.1.351 (hCoV-19/Korea/KDCA55905/2021 (NCCP 43382), Gamma P.1 (hCoV-19/Korea/KDCA95637/2021 (NCCP 43388), Delta B.1.617.2 (hCoV-19/Korea/KDCA119861/2021 (NCCP 43390), and Omicron B.1.1.529 (hCoV-19/Korea/KDCA447321/2021 NCCP43408) strains. Source data are provided as a Source Data file.

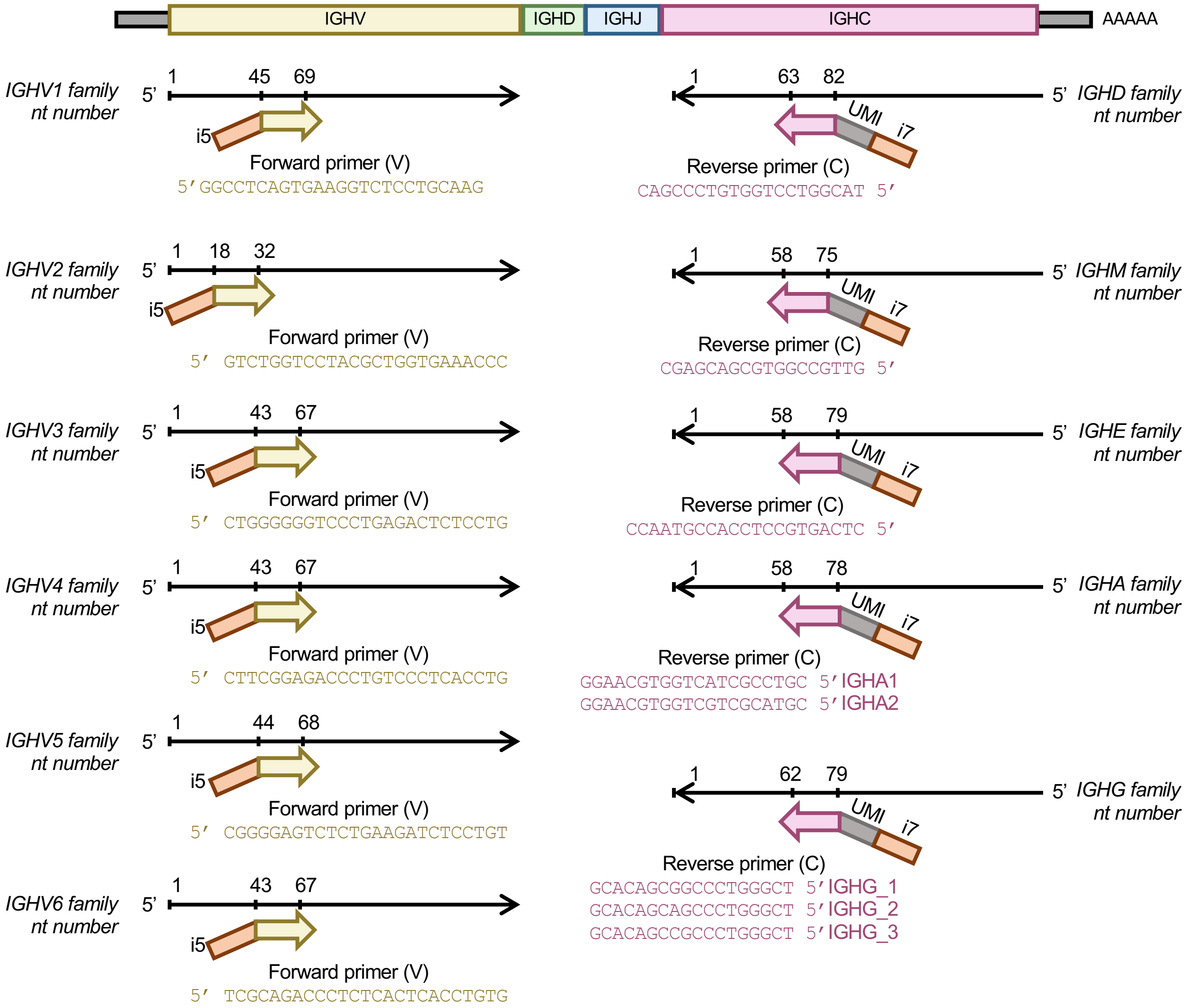

Supplementary Fig. 5 Overview of constructing the BCR HC libraries using gene specific primers

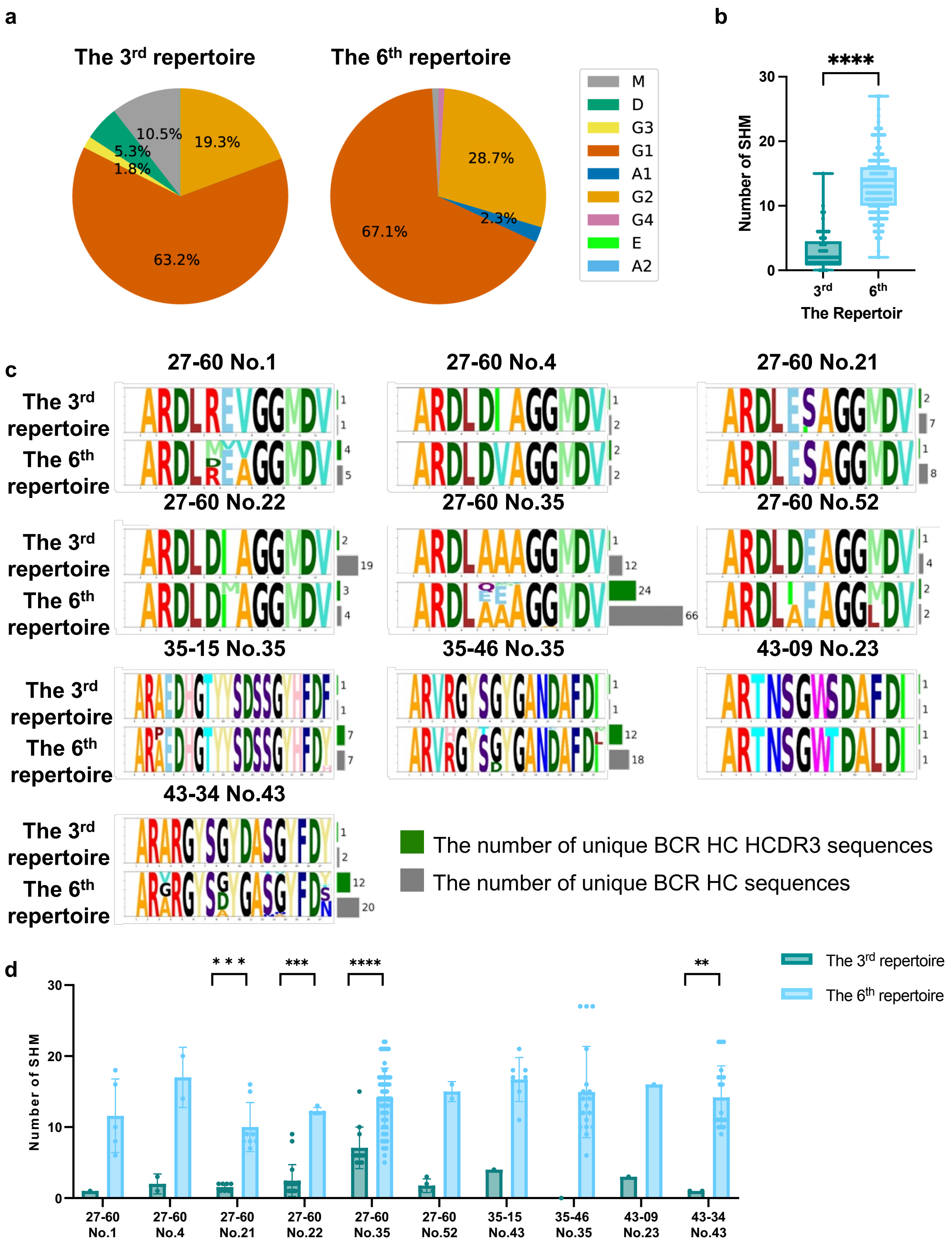
a
The 3rd repertoire
The 6th repertoire
10.5%
19.3%
5.3%
1.8%
63.2%
28.7%
2.3%
67.1%
M
D
G3
G1
A1
G2
G4
E
A2
b
****
Number of SHM
3rd
6th
The Repertoir
c
27-60 No.1
27-60 No.4
27-60 No.21
27-60 No.22
27-60 No.35
27-60 No.52
35-15 No.35
35-46 No.35
43-09 No.23
43-34 No.43
The 3rd repertoire
The 6th repertoire
The number of unique BCR HC HCDR3 sequences
The number of unique BCR HC sequences
d
Number of SHM
***
***
****
**
The 3rd repertoire
The 6th repertoire
27-60 No.1
27-60 No.4
27-60 No.21
27-60 No.22
27-60 No.35
27-60 No.52
35-15 No.43
35-46 No.35
43-09 No.23
43-34 No.43

Supplementary Fig. 6 The characteristics of the BCR HC sequences of nine BA.1RBD-reactive clonotypes mapped to the third and sixth repertoires. a, The isotype compositions of the BCR HC sequences in the third and sixth repertoires (n = 57 and 216, respectively). **b,** Numbers of SHMs in BCR HC sequences at the third and sixth repertoires (n = 57 and 216, respectively). The P value was calculated using a two-tailed independent t test. ****, $p < 0.0001$. **c,** Diversification of BCR HC sequences occurred from the third to the sixth repertoire through SHM and HCDR3 sequence variation. The numbers of unique BCR HC sequences and HCDR3 sequences are counted at the nucleotide level. **d,** Numbers of SHMs in the BCR HC sequences of clonotypes found in the third and sixth repertoires of the same vaccinee. The P value was calculated using the Mann–Whitney U test. In the case of repertoires for which statistical significance is not indicated, statistical significance was not calculated, as the number of clones included in the third or sixth repertoire was only one or two. ns, not significant, $p > 0.05$; *, $p < 0.05$; **, $p < 0.01$; ***, $p < 0.001$; ****, $p < 0.0001$. All box plots include the median line, the box denotes the interquartile range (IQR), whiskers denote the rest of the data distribution. Source data are provided as a Source Data file.

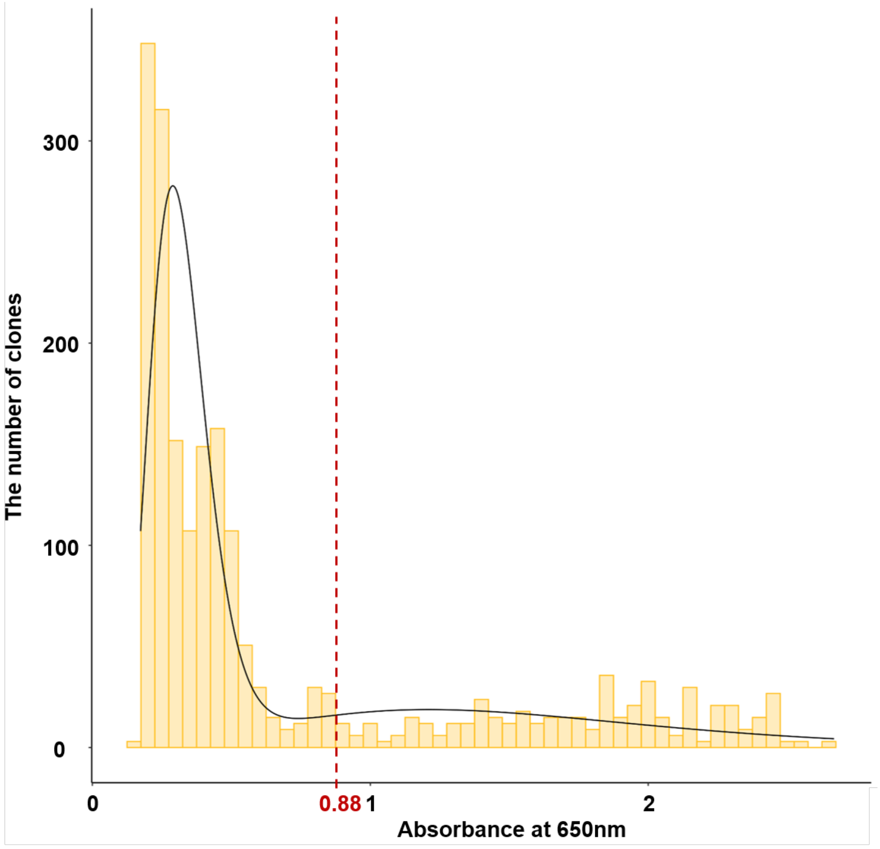

Supplementary Fig. 7 Determination of the threshold for RBD-reactive clones in the HCDR3-randomized scFv phage display library. After phage ELISA using both ancestral and BA.1 RBDs, the absorbance of each well was determined. The absorbance value was approximated with a Gaussian distribution by the binned kernel estimation method under the Gaussian kernel setting (black line). The chosen threshold absorbance value for a positive clone was 0.88 (red vertical line), which corresponded to the valley point in the distribution plot.

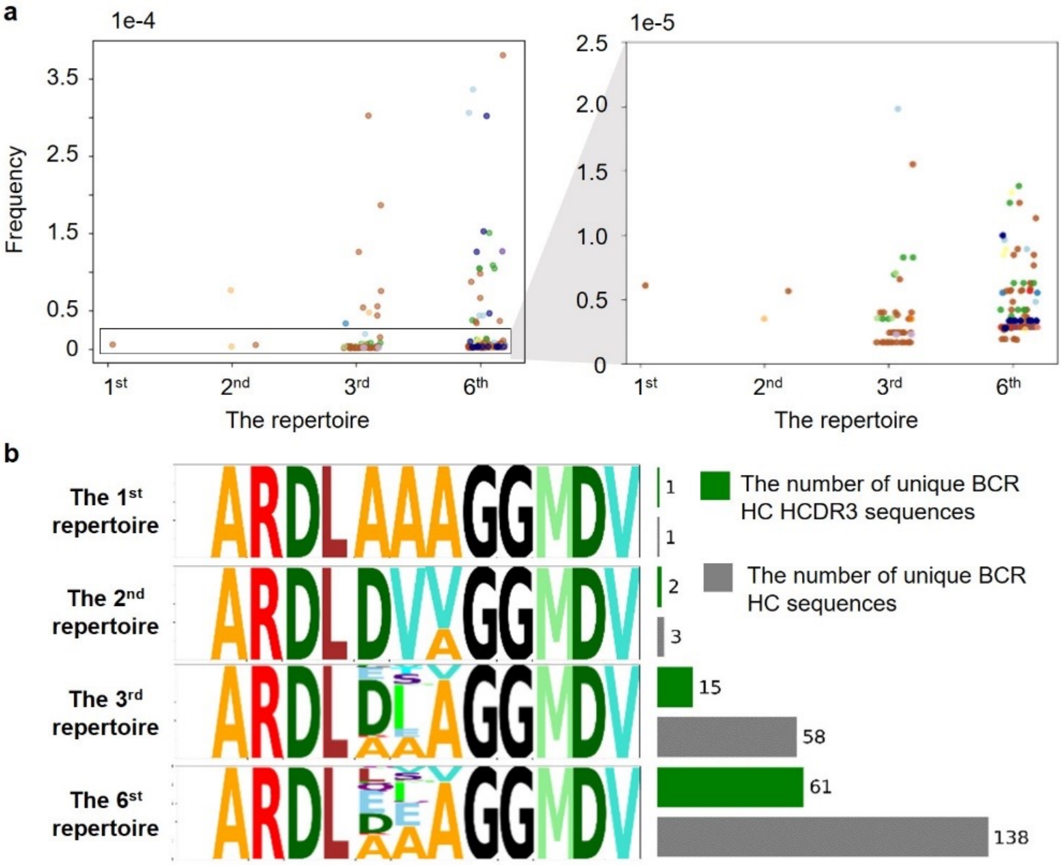

Supplementary Fig. 8 The frequency distribution and sequence diversity of BCR HC XXA/V clonotypes after the first, second, and third injections. a, The frequency distribution of BCR HC XXA/V clonotypes. The BCR HC XXA/V clonotypes with the same HCDR3 amino acid sequence are plotted in the same color. **b,** Diversity of BCR HC XXA/V sequences in individual repertoires. The numbers of unique BCR HC sequences and HCDR3 sequences are counted at the nucleotide level.

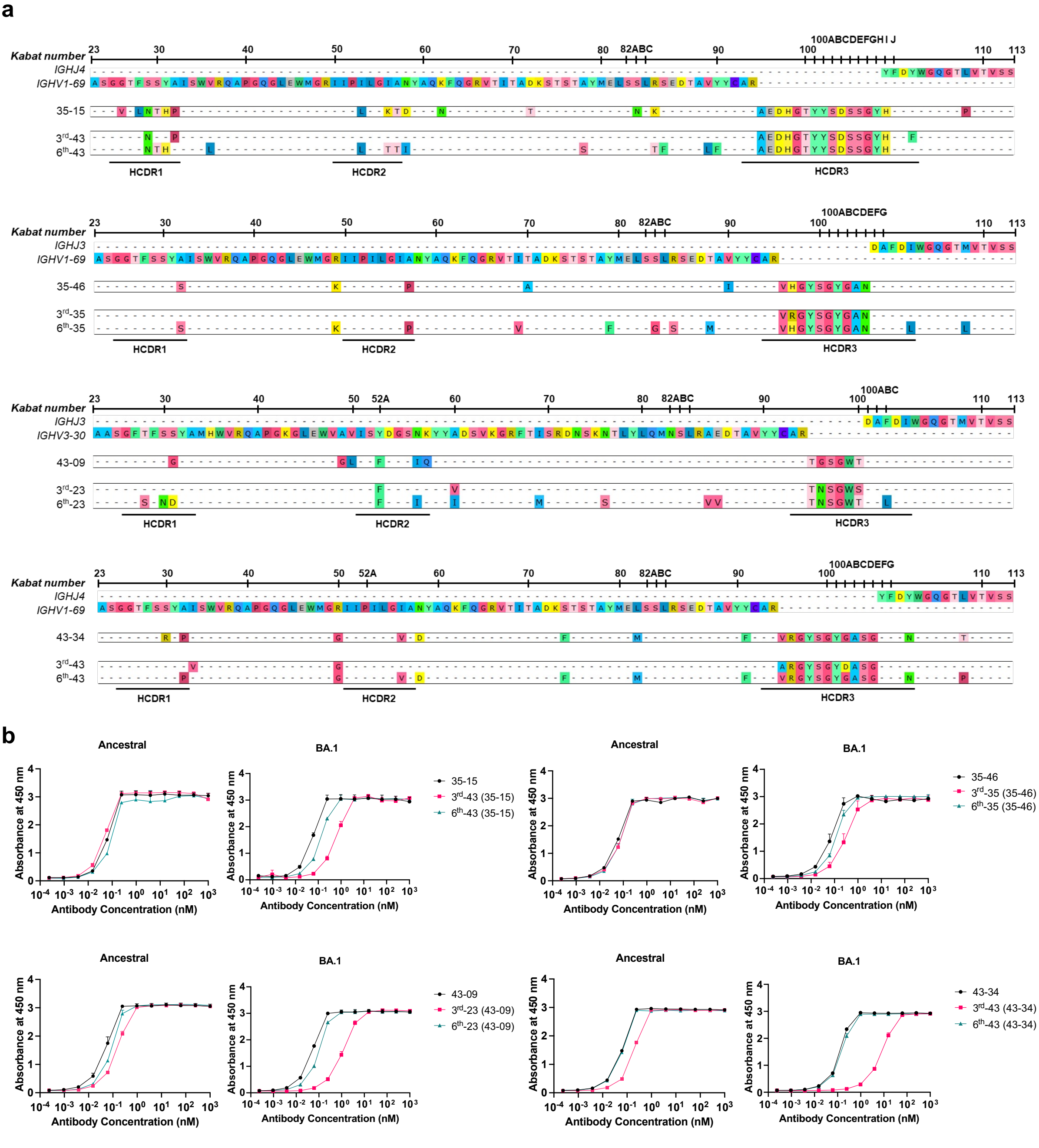

Supplementary Fig. 9 Characterization of the 35-15, 35-46, 43-09, and 43-34 BCR HC clonotype a, BCR HC sequences of four clonotypes found in vaccinees in their third and sixth BCR repertoires with the highest frequency. **b,** Reactivity of recombinant scFv-hFc-HA proteins encoded by individual BCR HC sequences and the light chain gene of each scFv clone to the ancestral and BA.1 RBDs. All experiments were performed in duplicate, and the data are presented as the means ± SD. Source data are provided as a Source Data file.

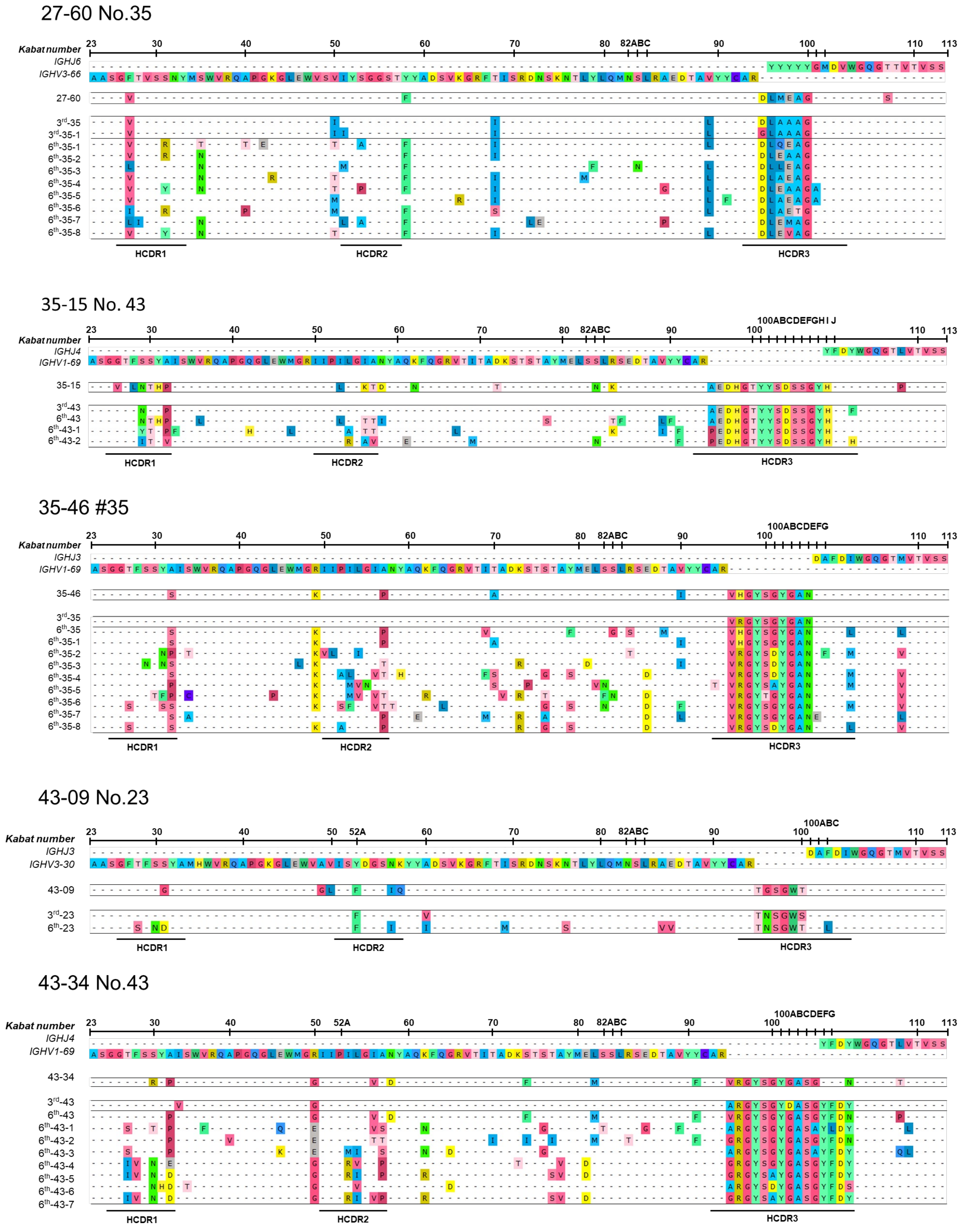

Supplementary Fig. 10 The BCR HC sequences of the 27-60, 35-15, 35-46, 43-09, and 43-34 clonotypes-3rd found in vaccinees 35, 43, 35, 23, and 43.

a

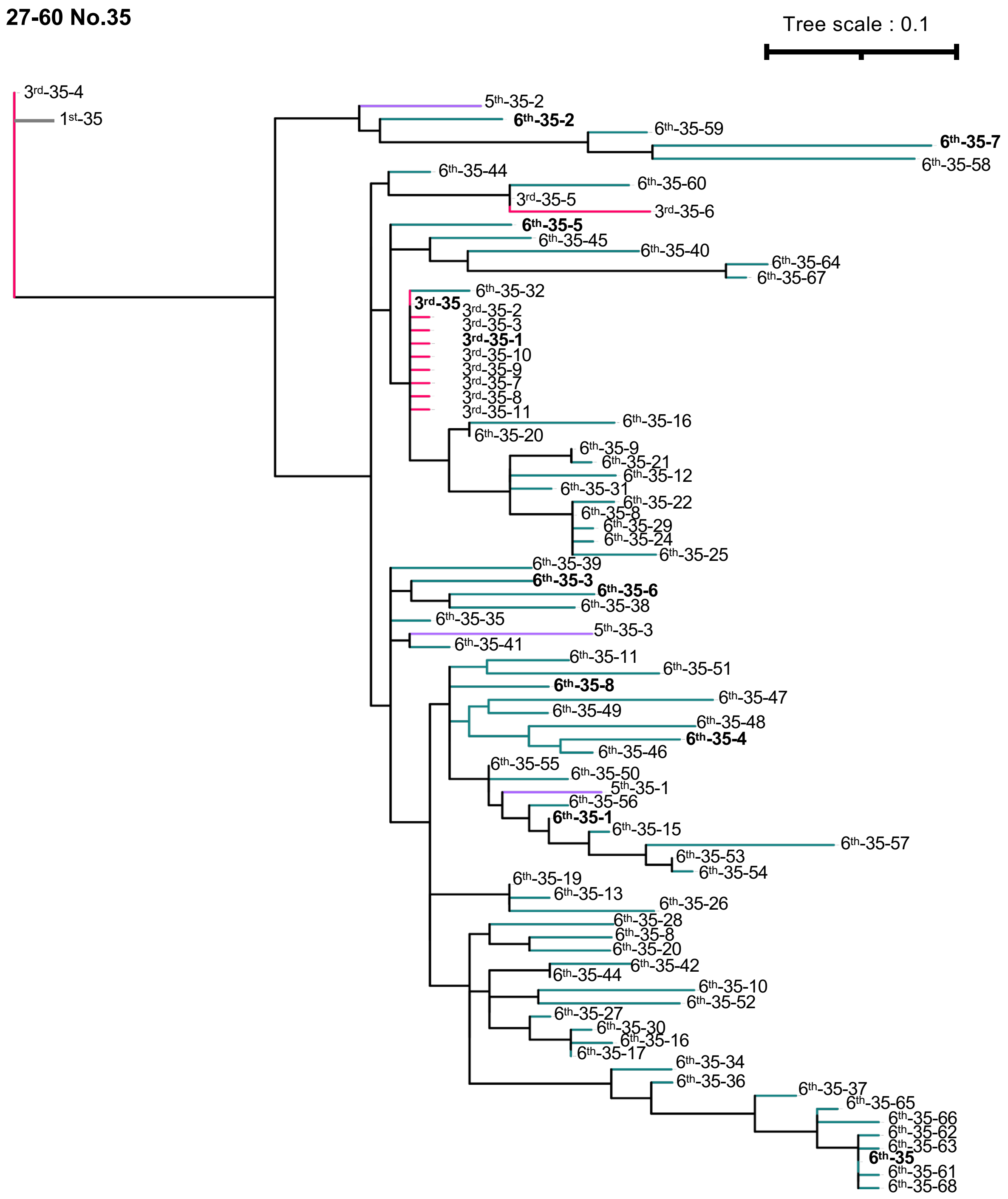
27-60 No.35
Tree scale : 0.1
3rd-35-4
1st-35
5th-35-2
6th-35-2
6th-35-59
6th-35-7
6th-35-58
6th-35-44
6th-35-60
3rd-35-5
3rd-35-6
6th-35-5
6th-35-45
6th-35-40
6th-35-64
6th-35-67
6th-35-32
3rd-35
3rd-35-2
3rd-35-3
3rd-35-1
3rd-35-10
3rd-35-9
3rd-35-7
3rd-35-8
3rd-35-11
6th-35-16
6th-35-20
6th-35-9
6th-35-21
6th-35-12
6th-35-31
6th-35-22
6th-35-8
6th-35-29
6th-35-24
6th-35-25
6th-35-39
6th-35-3
6th-35-6
6th-35-38
6th-35-35
5th-35-3
6th-35-41
6th-35-11
6th-35-51
6th-35-8
6th-35-47
6th-35-49
6th-35-48
6th-35-4
6th-35-46
6th-35-55
6th-35-50
5th-35-1
6th-35-56
6th-35-1
6th-35-15
6th-35-57
6th-35-53
6th-35-54
6th-35-19
6th-35-13
6th-35-26
6th-35-28
6th-35-8
6th-35-20
6th-35-42
6th-35-44
6th-35-10
6th-35-52
6th-35-27
6th-35-30
6th-35-16
6th-35-17
6th-35-34
6th-35-36
6th-35-37
6th-35-65
6th-35-66
6th-35-62
6th-35-63
6th-35
6th-35-61
6th-35-68

b

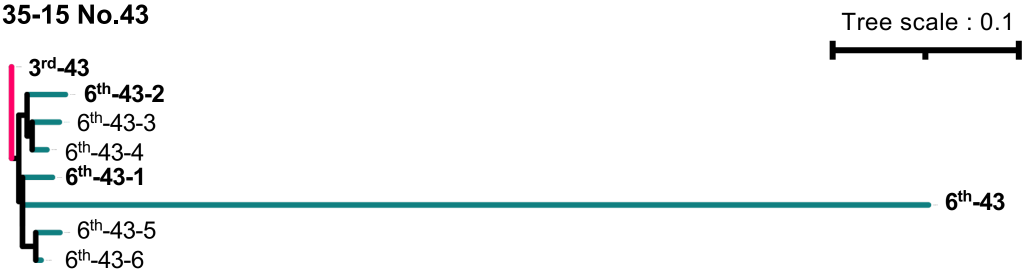
35-15 No.43
Tree scale : 0.1
3rd-43
6th-43-2
6th-43-3
6th-43-4
6th-43-1
6th-43
6th-43-5
6th-43-6

c

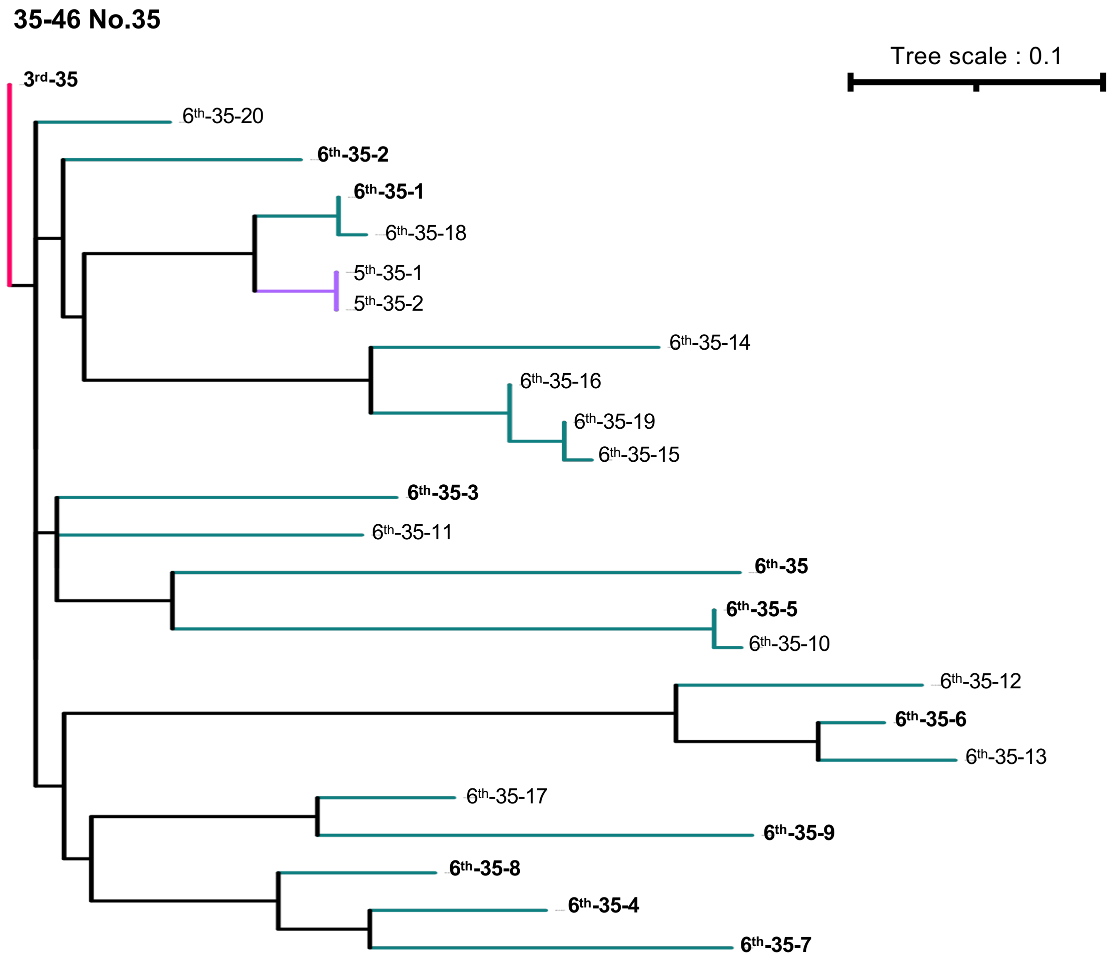
35-46 No.35
Tree scale : 0.1
3rd-35
6th-35-20
6th-35-2
6th-35-1
6th-35-18
5th-35-1
5th-35-2
6th-35-14
6th-35-16
6th-35-19
6th-35-15
6th-35-3
6th-35-11
6th-35
6th-35-5
6th-35-10
6th-35-12
6th-35-6
6th-35-13
6th-35-17
6th-35-9
6th-35-8
6th-35-4
6th-35-7

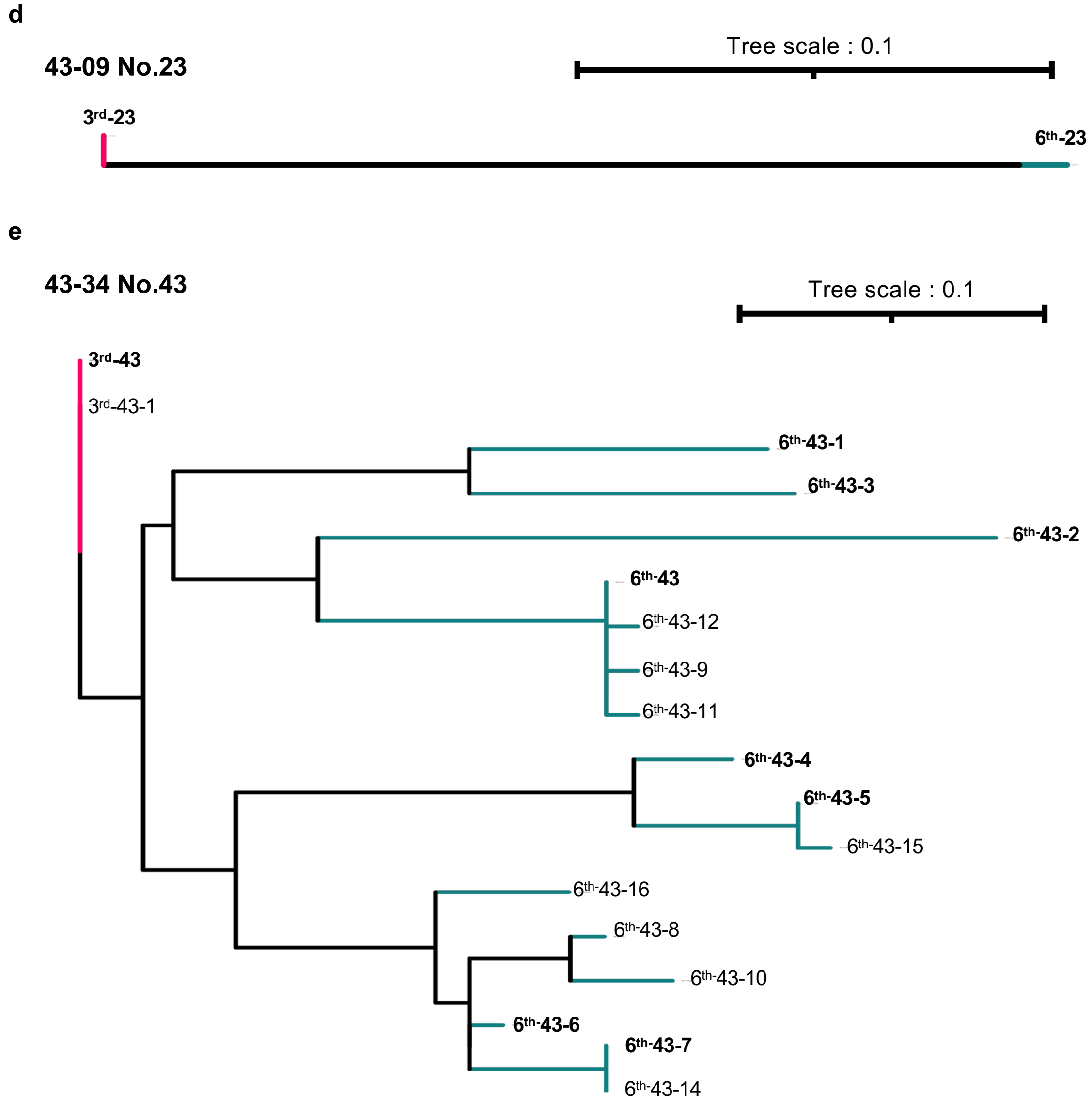

Supplementary Fig. 11 Phylogenetic trees of the 27-60, 35-15, 35-46, 43-09, and 43-34 clonotypes-3rd found in vaccinees 35, 43, 35, 23, and 43. The BCR sequences of clonotype-3rd in **(a)** 27-60 No.35, **(b)** 35-15 No.43, **(c)** 35-46 No.35, **(d)** 43-09 No.23, and **(e)** 43-34 No.43 that were synthesized to confirm the reactivity to ancestral RBD and those of Omicron subvariants are shown in bold. Source data are provided as a Source Data file.
