# Supplementary material for: An ancestral SARS-CoV-2 vaccine induces anti-Omicron variants antibodies by hypermutation
Source: Nat Commun. 2024 Apr 20;15:3368. doi: 10.1038/s41467-024-47743-1 (PMC11032360; doi:10.1038/s41467-024-47743-1)
Supplement: Supplementary file 3 — Description of Additional Supplementary Files [file 41467_2024_47743_MOESM3_ESM.pdf]

Description of Additional Supplementary Files

File name: Supplementary Data 1

Description: Vλ and VH sequences of the scFv clones of 27-60 cluster

File name: Supplementary Data 2

Description: BCR HC sequences of BA.1 RBD-reactive clonotypes

File name: Supplementary Data 3

Description: Characteristics of BA.1 RBD-reactive BCR HC clonotypes

File name: Supplementary Data 4

Description: CDR sequences of BA.1 RBD-reactive antibody clones

File name: Supplementary Data 5

Description: Demographic data of 41 vaccinees

File name: Supplementary Data 6

Description: Primers used in the study

File name: Supplementary Data 7

Description: The binding profile of the IGHV-specific forward primers to the IGHV allele germline sequences in IMGT database

File name: Supplementary Data 8

Description: The total read counts of the chronological repertoires after the NGS data processing

File name: Supplementary Data 9

Description: A total of 187 distinct IGHV alleles were identified across 246 BCR HC libraries at six time points from 41 vaccinees
