# Supplementary material for: An ancestral SARS-CoV-2 vaccine induces anti-Omicron variants antibodies by hypermutation
Source: Nat Commun. 2024 Apr 20;15:3368. doi: 10.1038/s41467-024-47743-1 (PMC11032360; doi:10.1038/s41467-024-47743-1)
Supplement: Supplementary file 5 — Reporting Summary [file 41467_2024_47743_MOESM5_ESM.pdf]

nature portfolio

Corresponding author(s): Seungtaek Kim, Myoung-don Oh, Sunghoon Kwon and Junho Chung

Last updated by author(s): Mar 28, 2024

Reporting Summary

Nature Portfolio wishes to improve the reproducibility of the work that we publish. This form provides structure for consistency and transparency in reporting. For further information on Nature Portfolio policies, see our Editorial Policies and the Editorial Policy Checklist.

Statistics

For all statistical analyses, confirm that the following items are present in the figure legend, table legend, main text, or Methods section.

n/a	Confirmed	
☐	☒	The exact sample size (n) for each experimental group/condition, given as a discrete number and unit of measurement
☐	☒	A statement on whether measurements were taken from distinct samples or whether the same sample was measured repeatedly
☐	☒	The statistical test(s) used AND whether they are one- or two-sided<br>*Only common tests should be described solely by name; describe more complex techniques in the Methods section.*
☒	☐	A description of all covariates tested
☒	☐	A description of any assumptions or corrections, such as tests of normality and adjustment for multiple comparisons
☐	☒	A full description of the statistical parameters including central tendency (e.g. means) or other basic estimates (e.g. regression coefficient) AND variation (e.g. standard deviation) or associated estimates of uncertainty (e.g. confidence intervals)
☐	☒	For null hypothesis testing, the test statistic (e.g. F, t, r) with confidence intervals, effect sizes, degrees of freedom and P value noted<br>*Give P values as exact values whenever suitable.*
☒	☐	For Bayesian analysis, information on the choice of priors and Markov chain Monte Carlo settings
☒	☐	For hierarchical and complex designs, identification of the appropriate level for tests and full reporting of outcomes
☒	☐	Estimates of effect sizes (e.g. Cohen's d, Pearson's r), indicating how they were calculated

Our web collection on statistics for biologists contains articles on many of the points above.

Software and code

Policy information about availability of computer code

Data collection	Data were collected as raw NGS data on the Illumina NovaSeq 6000 250PE.<br>ELISA data were acquired using the Multiskan Go microplate spectrophotometer.<br>Microneutralization data were measured with EVOS Digital Inverted Imaging System with 40X lens (AMG).
Data analysis	NGS data preprocessing was performed as described in a previous study (Kim, S. I. et al. Sci Transl Med 13 (2021).<br>Multiple sequence alignment (MSA) was performed using Clustal Omega program v1.2.4 and processed with Ugene software v1.16.2.<br>ELISA and microneutralization data were analyzed using GraphPad Prism software v6.<br>Phylogenetic trees were generated with IgPhyML v1.1.3 052020 and visualized using Interactive Tree Of Life (iTOL) online tool v6.<br>All custom scripts are available on GitHub (https://github.com/BiNEL-SNU/SARS-CoV-2).

For manuscripts utilizing custom algorithms or software that are central to the research but not yet described in published literature, software must be made available to editors and reviewers. We strongly encourage code deposition in a community repository (e.g. GitHub). See the Nature Portfolio guidelines for submitting code & software for further information.

Data

Policy information about availability of data

All manuscripts must include a data availability statement. This statement should provide the following information, where applicable:

- Accession codes, unique identifiers, or web links for publicly available datasets
- A description of any restrictions on data availability
- For clinical datasets or third party data, please ensure that the statement adheres to our policy

All sequencing data are available from the National Center for Biotechnology Information (www.ncbi.nlm.nih.gov/) under accession number PRJNA945512 (SRA). This study used data from the Coronavirus Antibody Database, CoV-AbDab (https://opig.stats.ox.ac.uk/webapps/covabdab/).

Research involving human participants, their data, or biological material

Policy information about studies with human participants or human data. See also policy information about sex, gender (identity/presentation), and sexual orientation and race, ethnicity and racism.

Reporting on sex and gender	The study involved a total of 41 participants, consisting of 19 males and 22 females. In the study design, sex was not taken into consideration, and we did not conduct a sex analysis as we were not interested in investigating differences in the BCR repertoire based on sex.
Reporting on race, ethnicity, or other socially relevant groupings	All participants in the study were of Korean descent and were health care workers employed at Seoul National University Hospital.
Population characteristics	Participants were healthy volunteers receiving third doses of Pfizer-BioNTech (BNT162b2) mRNA vaccines against SARS-CoV-2 who were recruited for serial blood donations at Seoul National University Hospital in Korea between March 5 and December 27, 2021. The vaccinees had a median age of 30 years (range 23-62) and showed a nearly equal distribution of males and females (46% and 54%, respectively).
Recruitment	The study recruited healthy healthcare workers scheduled for COVID-19 vaccination. Potential self-selection and recruiting biases are unlikely to affect the parameters we measured. All participants provided written informed consent before study participation and were requested to provide information on their health status and medications prior to the start of the clinical study. Participants received compensation for transportation expenses upon their visit for blood collection.
Ethics oversight	Institutional Review Board (IRB) at the Seoul National University Hospital, 2102-032-1193.

Note that full information on the approval of the study protocol must also be provided in the manuscript.

Field-specific reporting

Please select the one below that is the best fit for your research. If you are not sure, read the appropriate sections before making your selection.

☒ Life sciences ☐ Behavioural & social sciences ☐ Ecological, evolutionary & environmental sciences

For a reference copy of the document with all sections, see nature.com/documents/nr-reporting-summary-flat.pdf

Life sciences study design

All studies must disclose on these points even when the disclosure is negative.

Sample size	We collected blood samples from 41 BNT162b2 vaccinees following the course of three injections and analyzed their B-cell receptor (BCR) repertoires at six time points in total. The sample size of vaccinees was not predetermined using a statistical method but rather chosen based on the feasibility of enrolling participants during the enrollment period. Enrolling this sample size provided sufficient statistical power to analyze the effect sizes of interest.
Data exclusions	No data was excluded from the analyses.
Replication	To verify the reproducibility of our findings, ELISA and microneutralization assays were replicated at least for two and four times, respectively. All attempts to replication were successful.
Randomization	This is not applicable, as this is an observational study.
Blinding	This is not applicable, as this is an observational study.

Reporting for specific materials, systems and methods

We require information from authors about some types of materials, experimental systems and methods used in many studies. Here, indicate whether each material, system or method listed is relevant to your study. If you are not sure if a list item applies to your research, read the appropriate section before selecting a response.

Materials & experimental systems

n/a	Involved in the study
☐	☒ Antibodies
☐	☒ Eukaryotic cell lines
☒	☐ Palaeontology and archaeology
☒	☐ Animals and other organisms
☒	☐ Clinical data
☒	☐ Dual use research of concern
☒	☐ Plants

Methods

n/a	Involved in the study
☒	☐ ChIP-seq
☒	☐ Flow cytometry
☒	☐ MRI-based neuroimaging

Antibodies

Antibodies used

1. Goat anti-Human IgM-Peroxidase (Invitrogen, A18835)
2. Goat anti- Human IgA-Peroxidase (Invitrogen, A18781)
3. Rabbit anti-Human IgG-Peroxidase (Invitrogen, 31423)
4. Mouse anti-M13-Peroxidase (Sino Biological, 11973-MM05T-H)
5. Mouse anti-His antibody (Invitrogen, MA1-21315)
6. Rat anti-HA-Peroxidase (Roche, 12013819001)

Validation

All antibodies are commercially available and validated by manufacturers. Additionally information can be found on product website, listed below.
1. https://www.thermofisher.com/antibody/product/Goat-anti-Human-IgM-Heavy-chain-Secondary-Antibody-Polyclonal/A18835
2. https://www.thermofisher.com/antibody/product/Goat-anti-Human-IgA-Secondary-Antibody-Polyclonal/A18781
3. https://www.thermofisher.com/antibody/product/Rabbit-anti-Human-IgG-Fc-Secondary-Antibody-Polyclonal/31423
4. https://kr.sinobiological.com/antibodies/m13-11973-mm05t-h
5. https://www.thermofisher.com/antibody/product/6x-His-Tag-Antibody-clone-HIS-H8-Monoclonal/MA1-21315
6. https://www.sigmaaldrich.com/KR/ko/product/roche/12013819001

Eukaryotic cell lines

Policy information about cell lines and Sex and Gender in Research

Cell line source(s)

Monoclonal antibody expression: Expi293F™ Cells (Gibco, A14527)
Authentic virus microneutralizing assay: Vero Cells (ATCC, CCL-81)

Authentication

The cell lines were not authenticated.

Mycoplasma contamination

The cells were checked for mycoplasma contamination by Hoechst staining, and confirmed negative.

Commonly misidentified lines
(See ICLAC register)

No commonly misidentified cell lines were used.

Plants

Seed stocks

Report on the source of all seed stocks or other plant material used. If applicable, state the seed stock centre and catalogue number. If plant specimens were collected from the field, describe the collection location, date and sampling procedures.

Novel plant genotypes

Describe the methods by which all novel plant genotypes were produced. This includes those generated by transgenic approaches, gene editing, chemical/radiation-based mutagenesis and hybridization. For transgenic lines, describe the transformation method, the number of independent lines analyzed and the generation upon which experiments were performed. For gene-edited lines, describe the editor used, the endogenous sequence targeted for editing, the targeting guide RNA sequence (if applicable) and how the editor was applied.

Authentication

Describe any authentication procedures for each seed stock used or novel genotype generated. Describe any experiments used to assess the effect of a mutation and, where applicable, how potential secondary effects (e.g. second site T-DNA insertions, mosiacism, off-target gene editing) were examined.
